# Double impact: natural molluscicide for schistosomiasis vector control also impedes development of *Schistosoma mansoni* cercariae into adult parasites

**DOI:** 10.1371/journal.pntd.0005789

**Published:** 2017-07-28

**Authors:** Ronaldo de Carvalho Augusto, Guillaume Tetreau, Philippe Chan, Marie-Laure Walet-Balieu, Clélia Christina Mello-Silva, Claudia Portes Santos, Christoph Grunau

**Affiliations:** 1 Laboratório de Avaliação e Promoção da Saúde Ambiental, Instituto Oswaldo Cruz, Fundação Oswaldo Cruz, Brasil; 2 Univ. Perpignan Via Domitia, IHPE UMR 5244, CNRS, IFREMER, Univ. Montpellier, Perpignan, France; 3 PISSARO Proteomic Platform, Institute for Research and Innovation in Biomedicine, University of Rouen, Rouen, France; George Washington University School of Medicine and Health Sciences, UNITED STATES

## Abstract

**Background:**

Schistosomiasis has been reported in 78 endemic countries and affects 240 million people worldwide. The digenetic parasite *Schistosoma mansoni* needs fresh water to compete its life cycle. There, it is susceptible to soluble compounds that can affect directly and/or indirectly the parasite’s biology. The cercariae stage is one of the key points in which the parasite is vulnerable to different soluble compounds that can significantly alter the parasite’s life cycle. Molluscicides are recommended by the World Health Organization for the control of schistosomiasis transmission and *Euphorbia milii* latex is effective against snails intermediate hosts.

**Methodology/Principal findings:**

We used parasitological tools and electron microscopy to verify the effects of cercariae exposure to natural molluscicide (*Euphorbia milii* latex) on morphology, physiology and fitness of adult parasite worms. In order to generate insights into key metabolic pathways that lead to the observed phenotypes we used comparative transcriptomics and proteomics.

**Conclusions/Significance:**

We describe here that the effect of latex on the adult is not due to direct toxicity but it triggers an early change in developmental trajectory and perturbs cell memory, mobility, energy metabolism and other key pathways. We conclude that latex has not only an effect on the vector but applies also long lasting schistosomastatic action. We believe that these results are of interest not only to parasitologists since it shows that natural compounds, presumably without side effects, can have an impact that occurred unexpectedly on developmental processes. Such collateral damage is in this case positive, since it impacts the true target of the treatment campaign. This type of treatment could also provide a rational for the control of other pests. Our results will contribute to enforce the use of *E*. *milii* latex in Brazil and other endemic countries as cheap alternative or complement to mass drug treatment with Praziquantel, the only available drug to cure the patients (without preventing re-infection).

## Author summary

Intestinal schistosomiasis is among the most important parasitic disease caused by helminthes, affecting 67 million people worldwide. Vector and intermediate host of the parasitic worm are fresh water snails. WHO recommends use of molluscicides for control of local transmission. Among those, natural plant extracts such as *Euphorbia milii* latex have attracted particular attention since they are sustainable and cheap. We had anecdotic evidence that *E*. *milii* latex also impacts infection outcome if treated snails were infected with *S*. *mansoni*. We show here that transient exposure of the human dwelling larvae (cercariae) to the latex at doses that do not affect its infectivity has effects 60 days later on the morphology, physiology and fitness of the adult parasite worms. In order to generate insights into key metabolic pathways that lead to the observed phenotypes we used comparative transcriptomics and proteomics. We show that the effect of latex on the adult is not due to direct toxicity but it triggers an early change in developmental trajectory and perturbs cell memory, mobility, energy metabolism and other key pathways. We conclude that latex has not only an effect on the vector but applies also long lasting schistosomastatic action. The present work might also provide insights on targets with implications for developing new interventions for schistosomiasis control.

## Introduction

Schistosomiasis is a chronic parasitic disease caused by trematodes of the *Schistosoma* genus. It has been reported in 78 endemic countries and affects more than 240 million people worldwide [1]. If left untreated, it is associated with fibrosis, abdominal pain, diarrhea and anemia, resulting in disabling patient symptoms [2]. The main symptoms of this disease are caused by the body's reaction to the parasites' eggs. In the case of intestinal schistosomiasis, caused by *Schistosoma mansoni*, the liver is the main target that can undergo enlargement frequently associated with an accumulation of fluid in the peritoneal cavity and hypertension of the abdominal blood vessels [2].

Controlling or preventing morbidity in patients has not been a very successful strategy to limit schistosomiasis transmission in high-risk areas. Optimal disease prevention can occur only when parasite infection and/or reinfection is effectively impeded [3]. In this sense, the World Health Organization (WHO) published a report of the WHO Strategic and Technical Advisory Group for Neglected Tropical Diseases (NTD) [4]. It addresses schistosomiasis management through the ecological control of population of the intermediate host of the parasite, snails from the *Biomphalaria* genus [4]. Molluscicides have been the primary method used for controlling schistosomiasis transmission. They can be divided into two classes: chemical and phytochemical compounds [5]. Among the chemical compounds, niclosamide is recommended by the WHO as the only chemical molluscicide to be used for snail control despite reported cases of resistance in mollusks after two decades of repeated use [6]. Many plants were tested as a source of potential phytochemical molluscicides [7,8,9]. *Euphorbia milii* var. *hislopii* was described as the most promising plant molluscicide [10]. Its latex exhibits molluscicidal activity at doses under 0.5 ppm in laboratory condition, it can be easily cultivated in endemic areas, it is biodegradable and it has been proved to be less damaging to non-target organisms than niclosamide, meeting the requirements of WHO for use as a natural molluscicide [2, 11]. A field study in Brazil was conducted resulting in disappearance of *B*. *glabrata* up to 14 month after two applications of 12 mg.L^-1^ of *E*. *milii* latex [12]. However, the use of a lower dose has been proposed as a promising method to control schistosomiasis transmission by the selective control of infected snails [13]. Although snails are very sensitive to latex, the effect of latex on cercaria survival and penetration are concentration-dependent and time-dependent, with no effect observed for exposure up to 8 mg.L^-1^ for 2 hours [14,15].

Cercariae of digenetic trematodes have complex structure characterized by a sequence of remarkable morphological and biochemical transitions between the aquatic environments to mammalian hosts [16]. They must swim to find their specific hosts before energy resources are exhausted. However, many soluble compounds and different forms of pollution can disrupt their interaction with the next host. The acquisition of soluble macromolecules through the tegument or by ingestion can induce changes in the gene expression in cercaria, which can affect its growth and development in its definitive host [17]. After infection, the success of host-parasite relationship depends, among other factors, on the expression, interaction and modulation of genes and proteins for the co-existence of both organisms [18]. In adult parasites, the transcriptome shows intense expression of genes mostly linked to the escape from the host immune system, to motility and to energy metabolism [16]. Besides that, the influence of environmental factors inside the host can also produce changes in the parasite gene expression and these could be mitotically heritable from schistosomula to adult worms [19]. Environmental factors can therefore affect some key genes and proteins that have consequences on parasite fitness, ultimately leading to a modification of disease dynamic and morbidity, which can make them good therapeutic targets for schistosomiasis control.

In the present work, the effect of *E*. *milii* latex on *S*. *mansoni* was investigated through a integrative multidisciplinary approach. After exposure of cercaria to latex at a low dose of 1.4 mg.L^-1^ in water, different parameters were measured in worms at their adult stage such as development in the murine host, morphology by scanning electron microscopy and granuloma reaction in the liver. Two month after the transient pulse treatment of cercariae with latex we found striking changes in phenotype and fitness of adult parasites. In order to provide insights into key metabolic pathways that could explain the observed phenotypes, both comparative transcriptomics and proteomics were used. These two approaches revealed effects on transcripts and proteins involved in parasite mobility, energy metabolism and other key pathways. We conclude that latex has a long lasting schistosomastatic effect and we hypothesize about the mechanisms that could be the bases of this effect.

## Methods

### Ethics

This research was approved by the Animal Ethics Committee of the Oswaldo Cruz Foundation (CEUA-FIOCRUZ LW-07/13) in agreement with the guidelines of the Brazilian College for Animal Experiments (COBEA). At IHPE, housing, feeding and animal care followed the national ethical standards established in the writ of February 1st, 2013 (NOR: AGRG1238753A). The French Ministère de l’Agriculture et de la Pêche and the French Ministère de l’Education Nationale de la Recherche et de la Technologie provided permit A66040 to the laboratory for animal experiments and certificate to the experimenters (authorization 007083, decree 87–848).

### Latex

The *E*. *milii* var. *hislopii* latex was collected at Ilha do Governador district (22°48´09´´S/43°12´35´´W), Rio de Janeiro, Brazil. The sample was pre-frozen in dry ice and absolute ethanol and subsequently lyophilized at -52°C on 8 x10^-1^ mBar for three 12-hour cycles in a Modulyo 4K Freeze Dryer with an acrylic chamber (Edwards High Vacuum Int., UK). The lyophilized pellet obtained with this process was diluted in distilled water and homogenized by sonication for 20 min. The dose of the powdered lyophilized latex of *E*. *milii* used to expose cercariae was 1.4 mg.L^-1^, described by [20] as LC_50_ for the intermediate host *Biomphalaria glabrata*.

### Parasites

The *S*. *mansoni* LE strain, originally sampled in Brazil was used in this study. Cercariae were collected from intermediate host *Biomphalaria glabrata* 30 days after infection by pipetting from spring water and sedimentation on ice and separated into two groups. The first group was exposed to a solution of *E*. *milii* lyophilized latex in distilled water (1.4 mg.L^-1^) for one hour. The second group (control) was kept in water for the same time period. Female mice were chosen as definitive host to avoid the effect of testosterone level in the parasite development [21]. We infected 57 female 4 weeks-old Swiss-Webster mice (weight mean: 18g) with 150 exposed cercariae per mouse (total of 8,550 exposed cercariae) and another 18 mice were infected with 150 mock treated cercariae per mouse (total of 2,700 control cercariae), all using standard percutaneous inoculation and mixed sexes [22]. Water and food were given *ad libitum*. Finally, parasites couples were recovered at 65 days post-infection by perfusion. Females and males were manually separated and counted. They were stored at room temperature in 70% ethanol for parasitological analysis and electronic microscopy and at -80°C for transcriptomic and proteomic analysis.

### Parasitological analysis

The intensity of infection and the reproduction of parasite female were measured by the Kato-Katz technique. This approach allows quantifying the number of eggs per gram of stool (EPG). For that, three slides in three different days were performed for each experimental group, which corresponds to the feces collected in a two-hour period between 10:00 and 12:00 a.m. The parasitological results were expressed as the mean number ± standard deviation and they were analyzed with Student's t test (α = 5%) performed using the R program (version 3.3.2, R Development Core Team, 2012). The graphics were constructed using GraphPad Prism software (GraphPad V.4.00, Prism, GraphPad, vol. 3.02, Prism Inc.).

### Histopathological analysis

The liver is the most important tissue for schistosomiasis disease considering that a high number of eggs and large granulomas are dangerous for liver healthy. To measure the intensity of hepatic granuloma inflammation, we fixed the entire liver in Milloning (37–40%formaldehyde, NaH_2_PO_4,_ NaOH, sucrose, pH 7.2–7.4) for subsequent histological examination in the same organ region. After successive washes with 70% ethanol to completely remove the fixative, tissues were dehydrated in an ethanol series from 70% to absolute alcohol. After this stage, the samples were cleared in xylene and embedded in histological paraffin melted at 60°C. Subsequently, they were embedded in paraffin, cut with a rotary microtome (Leica RM2125RT model, Nussloch—Germany), yielding sections of 5 μm thickness. The cuts intended for histopathology were stained with hematoxylin / eosin (HE) to visualize the granulomas and measure their size. We measured the diameter of the hepatic granulomas by Olympus CX31 microscope with 10X objective. On average, 35 granulomas were measured in each group analyzed. Statistical analyses were performed with Student's t test (α = 5%) using the R program (version 3.3.2, R Development Core Team, 2012).

### Ultrastructural analysis

To explore the ultrastructure of adult parasites resulting from latex-exposed or unexposed cercariae, we used scanning electron microscopy (SEM). To perform the SEM, the parasites were fixed in 2.5% glutaraldehyde in 0.1 M cacodylate buffer (pH 7.2). They were then washed in PBS (pH 7.2) and post-fixed in 1% osmium tetroxide in 0.1 M cacodylate buffer for 1 hour at room temperature in darkness. The dehydration step was performed in increasing concentrations of acetone before the critical point using CO_2_. The specimens were sputtered with gold and observed in a JEOL JSM 6390 at the Plataforma de Microscopia Eletrônica of Fiocruz, Brazil.

### Transcriptional profiling

To investigate latex-induced gene expression modifications, we performed RNA-Seq on female and male adult worms from each experimental group as described by [19]. Two biological replicates from each group were analyzed and each replicate consisted in 50 worms crushed in liquid nitrogen for 5 minutes. Total RNA was extracted by following TRIzol-Choroform extraction procedure [19]. RNA was purified by using PureLink RNA Mini kit (Ambion) following the manufacturer’s protocol and then eluted in 30 μL RNAsecure (Ambion). Each sample was then treated with TURBODNase (TURBODNA-free, Ambion), RNA was purified by using columns from RNeasy mini kit (QIAGEN) and eluted in 30 μL RNase-free water. Quality and concentration of RNA were assessed by spectrophotometry with the Agilent 2100 Bioanalyzer system.

### Illumina library generation and sequencing

Total RNA was quantified by NanoDrop Spectrophotometer ND-1000 (NanoDrop Technologies, Inc.) and its integrity was assessed by 2100 Bioanalyzer (Agilent Technologies). Libraries were generated from 250 ng of total RNA using the TruSeq stranded mRNA Sample Preparation Kit (Illumina), as per the manufacturer’s recommendations. Libraries were quantified using the Quant-iT PicoGreen dsDNA Assay Kit (Life Technologies) and the Kapa Illumina GA with Revised Primers-SYBR Fast Universal kit (Kapa Biosystems). Average size fragment was determined using a LabChip GX (PerkinElmer) instrument. The libraries were normalized, denatured in 0.05N NaOH and then were diluted to 8pM using HT1 buffer. The clustering was done on a Illumina cBot and the flowcell was ran on a HiSeq 2000 for 2x100 cycles following the manufacturer's instructions. A phiX library was used as a control and mixed with libraries at 1% level. The Illumina control software was HCS 2.2.58, the real-time analysis program was RTA v. 1.18.63. Program bcl2fastq v1.8.4 was then used to demultiplex samples and generate fastq reads.

### Transcriptomic profiling

For the analysis of global transcription mate-pair ended reads were aligned with STAR 2.4.0d [23] to the reference genome sma_v5.2.chr.fasta without gene model file for splice junctions. SAM attribute XS was added for and all non-canonical junctions were removed for downstream processing with cufflinks. For all other parameters (seed, alignment, and chimeric alignment) default values were used. For analysis of transcription from repetitive sequences paired-end reads were aligned using STAR with RepBasePerpignanSma52.fasta as reference repetome [24], no gene model file for splice junctions and length of the genomic sequence around annotated junctions set to 100. All other parameters were left as default.

Intron-exon structures were reconstructed with cufflinks v2.2.1 using Max Intron Length 300000, Min Isoform Fraction 0.1, Pre MRNA Fraction 0.15, without reference annotation, bias correction or multi-read correction [25]. Cufflinks effective length correction was used and for all other parameters default values. Cuffmerge 2.2.1.0 was used to combine cufflinks output files first separated by sex and then as a combined male and female transcription annotation in gtf format. BAM files were name sorted and Htseq-count version 0.6.0 in the Union mode, unstranded and Minimum alignment quality 10 Feature type exon and ID Attribute gene_id was used to obtain hit counts for every gene in the gtf combined cuffmerge file on both sexes [26]. For repeats, STAR alignements were converted into read counts with the galaxy tool sam2counts_edger version 1.0.0. ‘ = ‘ and ‘#’ were replaced in the sequence names for further processing. Differentially expressed genes and repeats were identified using DESeq2 version 1.8.2 (fit type parametric) under R version 3.2.1. Bonferroni adjusted -log10(P) ≥ 5 were considered as significant [27]. All analyses were done on the Galaxy instance of the IHPE (http://bioinfo.univ-perp.fr) [28].

### Proteomic profiling

To investigate latex-induced protein expression modifications, protein profiles were analyzed by 2D gel electrophoresis. Total proteins were extracted by UTC denaturing solution (7M urea, 2M thiourea, 30mM tris pH 8.5 and 4% CHAPS) of 5 adult male or 8 adult female parasites. Five biological replicates were processed for each sex and each group. Protein concentration of each sample was quantified using 2D Quant kit (GE Healthcare life sciences) and they were stored at -80°C until use. The samples were subjected to 2D gel electrophoresis using 100 μg of protein and a 17 cm ReadyStrip IPG Strips with a non-linear 3–10 pH gradient (BioRad). Isoelectric focusing was initiated immediately after sample loading on the strip by 5 h of passive rehydration followed by 14 h of active rehydration (50 V). Rehydration and focusing were both performed on a Protean IEF Cell system (Bio-Rad) at 20°C following a four-step program: 50 V for 1 h, 250 V for 1 h, 8,000 V for 1 h and a final step at 8,000 V for a total of 90,000 V.h with a slow ramping voltage (quadratically increasing voltage) at each step. After isoelectric focusing, the strips were reduced twice in equilibration buffer (6 M urea, 0.075 M Tris HCl (pH 8.8), 29.3% glycerol, 2% SDS, and 0.002% bromophenol blue) containing 2% dithiothreitol (DTT) and alkylated once in equilibration buffer containing 5% iodoacetamide.

For the second dimension, the strips were placed on a 12%/0.32% acrylamide/piperazine diacrylamide gel run at 25 mA/gel for 30 min followed by 75 mA/gel for 8 h using a Protean II XL system (Bio-Rad). Protein standards were loaded with whatman paper impregnated with 3 μL of Unstained Precision Plus Protein Standards (Bio-Rad) on the left part of the gels. Gels were stained using regular silver staining, and comparative analysis of digitized proteome maps was performed using the PDQuest 7.4.0 image analysis software (Bio-Rad). Only spots whose abundance was significantly different between exposed and control conditions at a significance level of p< 0.05 based on one-way ANOVA analysis (assuming equal variance) and a ratio above 1.5 were considered.

Selected spots were manually excised from MS-compatible silver stained gels using a Onetouch Plus Spot Picker Disposable (Harvard Apparatus) equipped with specific 1.5 mm methanol-washed tips. Gel plugs were first destained following a potassium ferricyanide/sodium thiosulfate procedure and then, proteins were trypsin-digested overnight at 30°C. Peptides were recovered after three washes a solution of formic acid (1%) and acetonitrile (50%). Peptides were lyophilized and sent to the PISSARO proteomic platform (University of Rouen, France) for identification with a nano-LC1200 system coupled to a Q-TOF 6550 mass spectrometer equipped with a nanospray source and an HPLC-chip cube interface (Agilent Technologies). For protein identification, peak lists were extracted (merge MSn scans with the same precursor at +/- 30 s retention time window and +/- 50 ppm mass tolerance) and compared with *S*. *mansoni* genome as reference database by using the PEAKS studio 7.5 proteomics workbench (Bioinformatics Solutions Inc., build 20150615). Only significant hits with a false discovery rate (FDR ≤ 1) for peptide and protein cut off (-logP ≥ 20 and unique peptides ≥ 2) were considered.

### Pathways analysis

To improve our understanding of the changes caused by latex exposure in both sexes, a pathway analysis combining transcripts and proteins differentially abundant for each sex and each exposure group was conducted. First of all, the biological functions of the transcripts/proteins differentially expressed in female and in male between control and exposed groups were annotated by Gene Ontology Annotation and AmiGO2. We focused our analysis on biological processes only and the number of GO terms in each category was analyzed for each parasite sex. To obtain a more synthetic overview, we used the highest hierarchical level as categories.

Additionally, to gain insight into predicted protein-protein interactions and affected pathways on female and male, enrichment analysis using String Database were performed by focusing on pathways containing genes and/or proteins that were under-expressed or over-expressed [29]. Only the highest confidence interactions (*i*.*e*., interactions with a score larger than 0.9) were considered, including direct (physical) and indirect (functional) associations derived from four different sources: “genomic context” (associations based on physical location of genes on genome that indicates that encoded proteins participates in the same metabolic pathway), “high throughput” (associations based on the function of proteins), “co-expression” (associations based on simultaneous expressions of different genes in the same organism) and “previous knowledge” (associations from previous annotations in other databases).

## Results

### Latex exposure of parasite worms at cercarial stage results in reduced fitness at the adult stage

**Fig 1:**
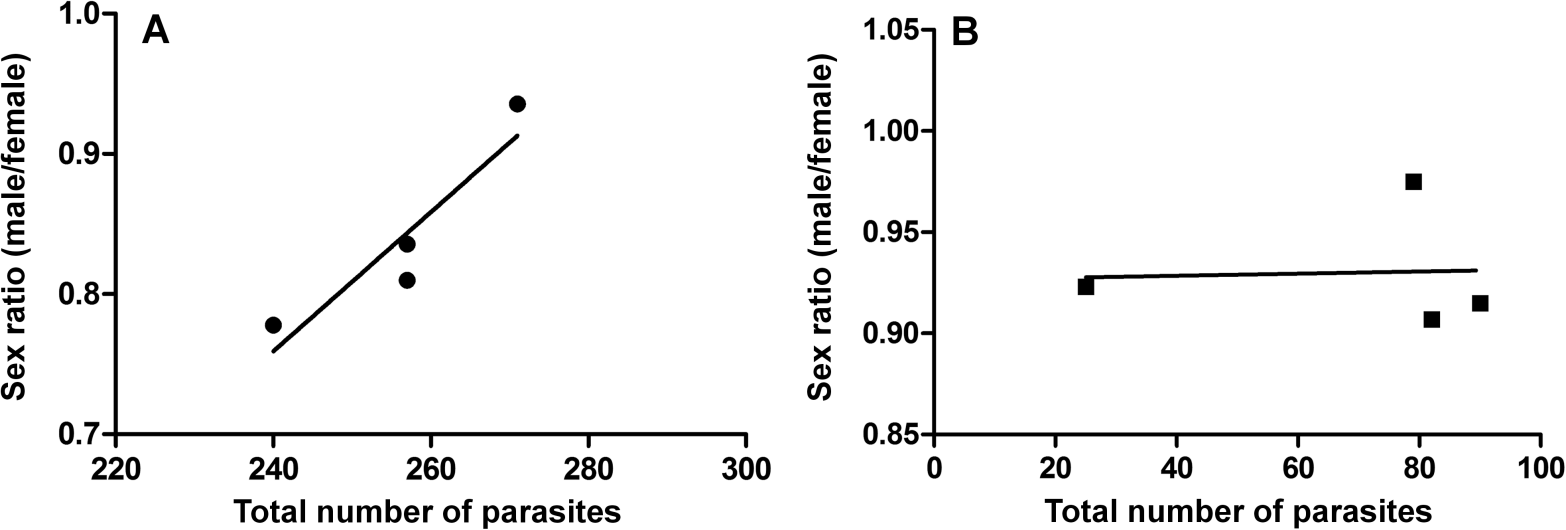
Sex ratio of *Schistosoma mansoni* (male parasites / female parasites) in function of parasite load. The parasite load was measured in 18 infected control mice and 57 mice infected with 150 exposed cercariae per mouse. (A) Parasite load in control group: linear regression R^2^ = 0.85, Y = 4.0 E^-3^x + 87.13; (B) Parasite load in Swiss Webster mouse infected with latex-exposed *S*. *mansoni*: linear regression R^2^ = 0.002, Y = 5.2 E^-5^x - 1.7752. Every point represents perfusion of worms from all mice of single cage (5-20 mice). The cages were also used for Kato-Katz.

In the exposed group, the number of adult parasites and the number of recovered couples were 37.7% and 46.3% lower compared to control parasites, respectively (Table 1). Sex ratio was changed with an increased proportion of *S*. *mansoni* males in the control population when the number of parasites increases (Fig 1A) but not in the latex-exposed group (Fig 1B). The quantity of eggs per female was 36.7% lower in the exposed group with a mean of 0.79 and 0.17 eggs per female in control and exposed groups, respectively (Student’s t test, *p* < 0.001) (Table 1). The significant reduction of the number of adult parasites (Student’s t test, *p* < 0.01) associated with a significant decreased number of eggs released per female caused a 89.2% reduction of the number of eggs recovered from mice (Student’s t test, *p* < 0.01) (Table 1).

**Table 1:** Effect of exposure to *Euphorbia milii* latex on the adult parasite fitness parameters. Control group was composed with of 18 mice infected with 150 mock treated cercariae per mouse and exposed group was composed of 57 mice infected with 150 exposed cercariae per mouse. All infections were performed by standard percutaneous inoculation and mixed sexes. Comparisons between groups were done by Student´s t-test.

	Adult parasites per mouse	Recovered couples per mouse	Eggs per female parasite	Egg released per mouse	Sex ratio (male/female)	Granuloma size (mm x 10^−3^)
**Control**	28.9±9.7	8.2±4.5	0.79±0.1	30.7±3.4	0.83±0.06	2.0±0.5
**Exposed**	18.0±11.5^*^	4.4±4.0^**^	0.17±0.1^**^	3.3±2.9^*^	0.93±0.03^*^	1.8±0.3^*^

Asterisks indicate statistically significant difference between control and exposed group in the same parameter

*P ≤ 0.01

** P ≤ 0.001).

**Fig 2:**
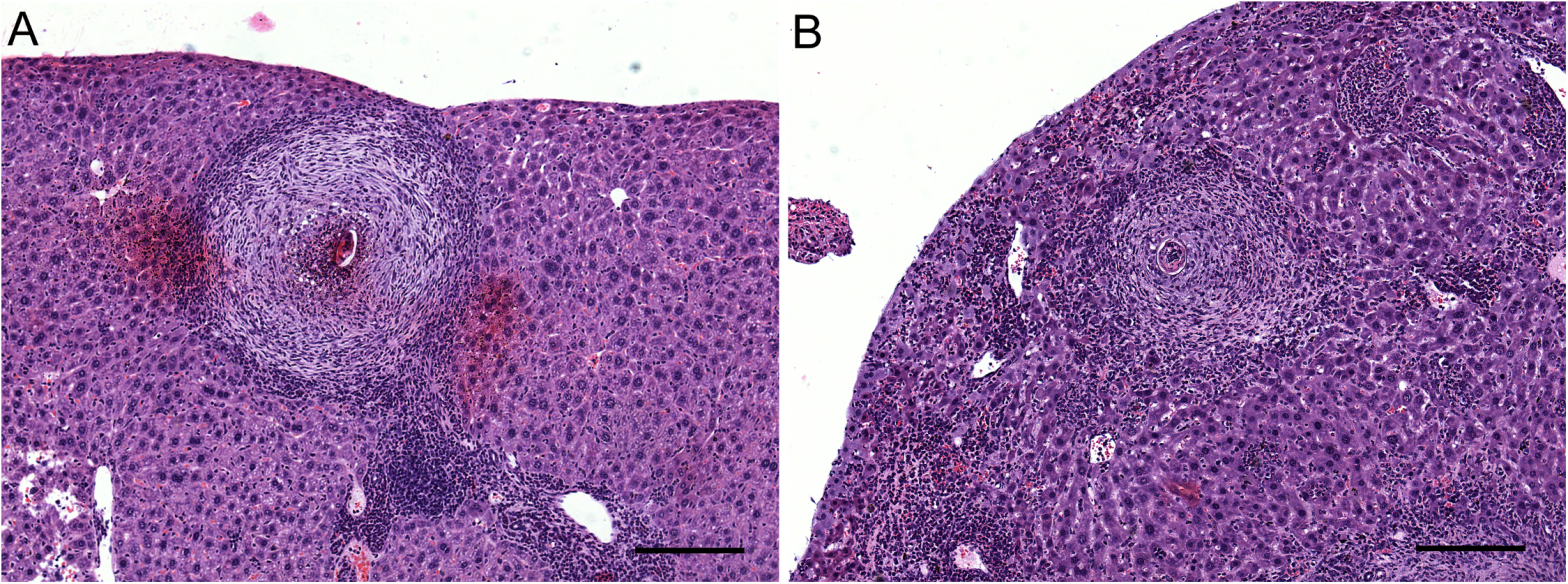
Hepatic granuloma of mice infected with *Schistosoma mansoni*. The samples were randomly chosen among 18 mice from control group and 57 mice from exposed group. (A) Hepatic granulomas in control group. (B): Hepatic granulomas in Swiss Webster mouse infected with latex-exposed *S*. *mansoni*. Bar: 200 μm.

Periovular granulomas were observed isolated and sparsely distributed in the hepatic parenchyma, sometimes forming clusters, but it was not possible to observe differences between groups. Moreover, in many of these granulomas a deposition of small amounts of collagen was noticed in many of these granulomas. Intense eosinophilic infiltration also occurred in medium and large portal spaces and sometimes in the central portion of the granulomas (Fig 2). The diameter of the hepatic granulomas in definitive hosts infected with cercariae exposed to latex was 10% smaller than those of control group (Student’s t test, *p* = 0.01) (Table 1).

### Latex exposure at cercarial stage leads to changes in the ultrastructure of the tegument of the adult worms

**Fig 3:**
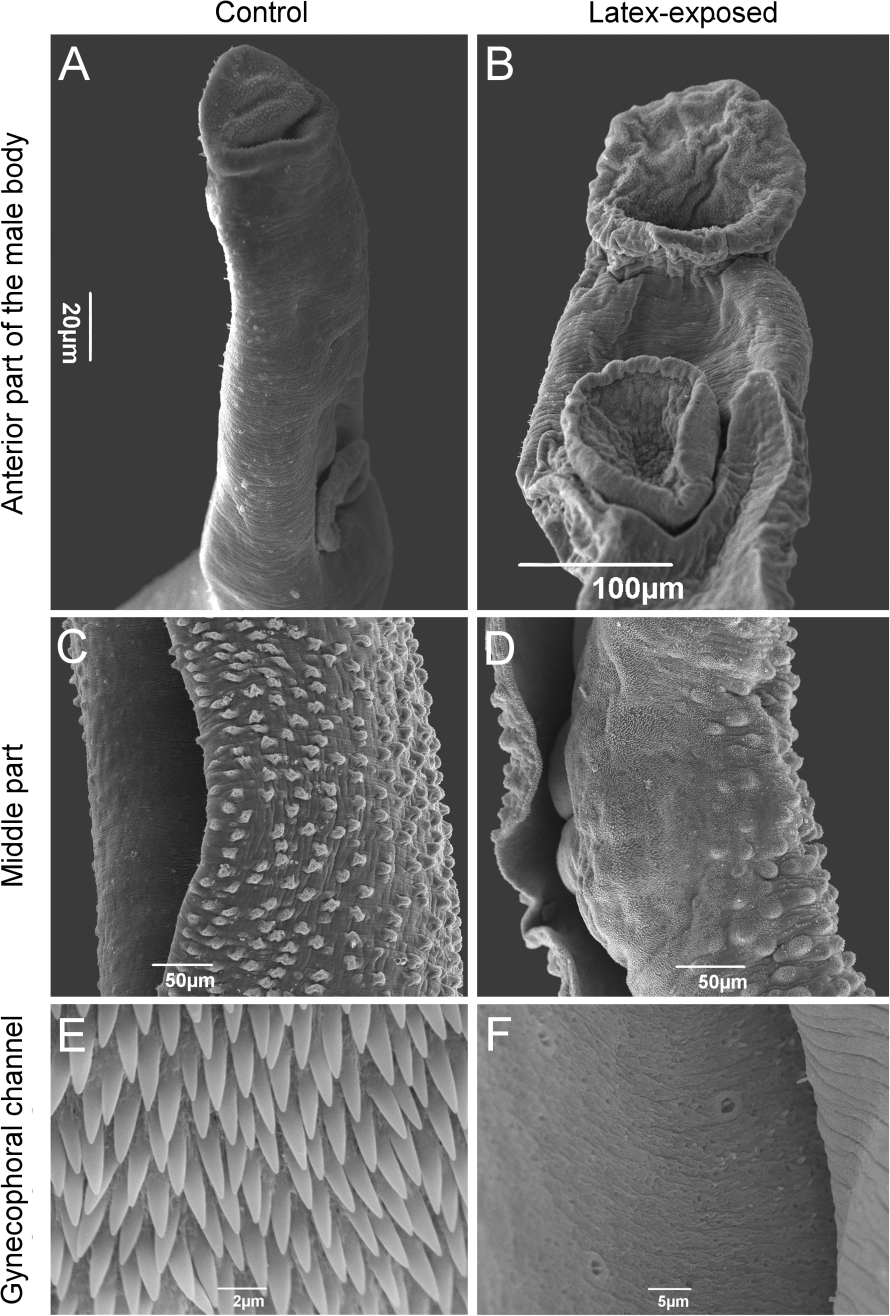
Scanning electron micrographs of adult male *Schistosoma mansoni*. Oral and ventral suckers of parasites from control (A) and latex-exposed groups (B); Tegument of worms from control (C) and latex-exposed groups (D); Gynecophoral channel of worms with spines from control (E) and without spines for latex-exposed ones (F).

**Fig 4:**
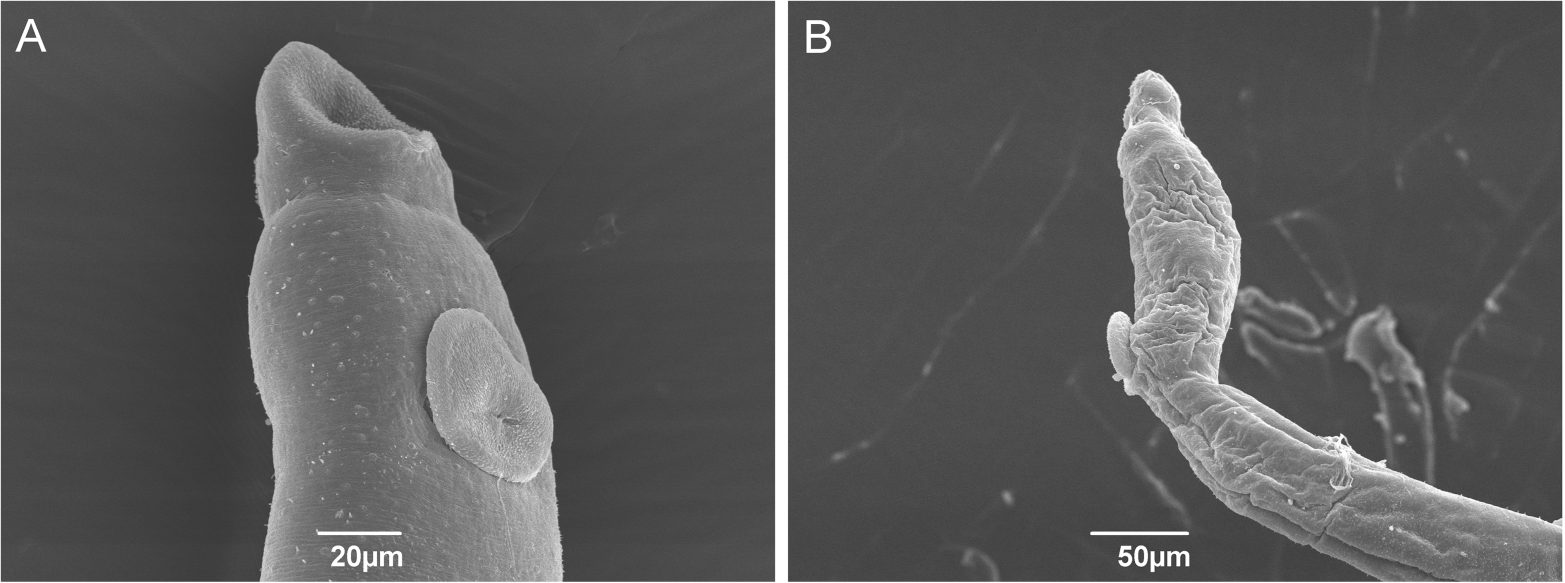
Scanning electron micrographs of adult female *Schistosoma mansoni*. Oral and ventral suckers of female parasites from control (A) and latex-exposed groups (B).

The ultrastructure of the mid-dorsal tegument of *S*. *mansoni* males exposed to latex exhibited pronounced changes as compared to control ones, with an important loss of pattern in the distribution of tubercles and spines on the mid-dorsal surface (Fig 3A–3D). In some areas, wrinkles and complete absence of tubercles and spines were observed (Fig 3C and 3D). The ultrastructure of the gynecophoral channel of male worms from exposed group revealed a complete loss of spines in the area as compared to control ones (Fig 3E and 3F). The females from exposed group exhibited remarkable wrinkles both in the tegument and suckers as compared to undamaged females from control (Fig 4).

### Thirty-nine genes show differences in RNA level in adults when cercariae had been exposed to latex

In total, the transcriptome sequencing of the two experimental groups yielded 1,080,386,261 Illumina single reads and 981,363,482 of them (90.8%) were mapped to the *S*. *mansoni* reference genome (v5.2). These mapped reads in female samples were reconstructed into 6,654 genes (XLOC) ans 9,598 unique transcripts (TCONs) (S1 Table). In male samples, they were 16,192 unique genes and 33,396 transcripts, respectively (S1 Table).

Quantification of read abundance and DEseq2 analysis of differential gene expression between sexes and experimental groups (adjusted *P*-value *<* 0.05) identified 24 genes that were under-expressed in female adults from the exposed group as compared to those from control group (S2 Table). Conversely, 15 genes were differentially expressed between exposed and control groups in male adult worms, with 11 upregulated in exposed group (S2 Table). No significant differences in mRNA levels were detected for repeats.

Among the differently expressed genes, several transcripts related to cell cycle control (Putative meiosis-specific nuclear structural protein 1, Putative microfibril-associated protein), neoblast formation (Bruno-like rna binding protein) and energy metabolism (Putative ATPase class VI and type 11c, Putative ribosome biogenesis protein BMS1, Ceramidase) were found.

### Forty-eight proteins showed differences in abundance in adults when cercariae had been treated with *E*. *milii* latex

**Fig 5:**
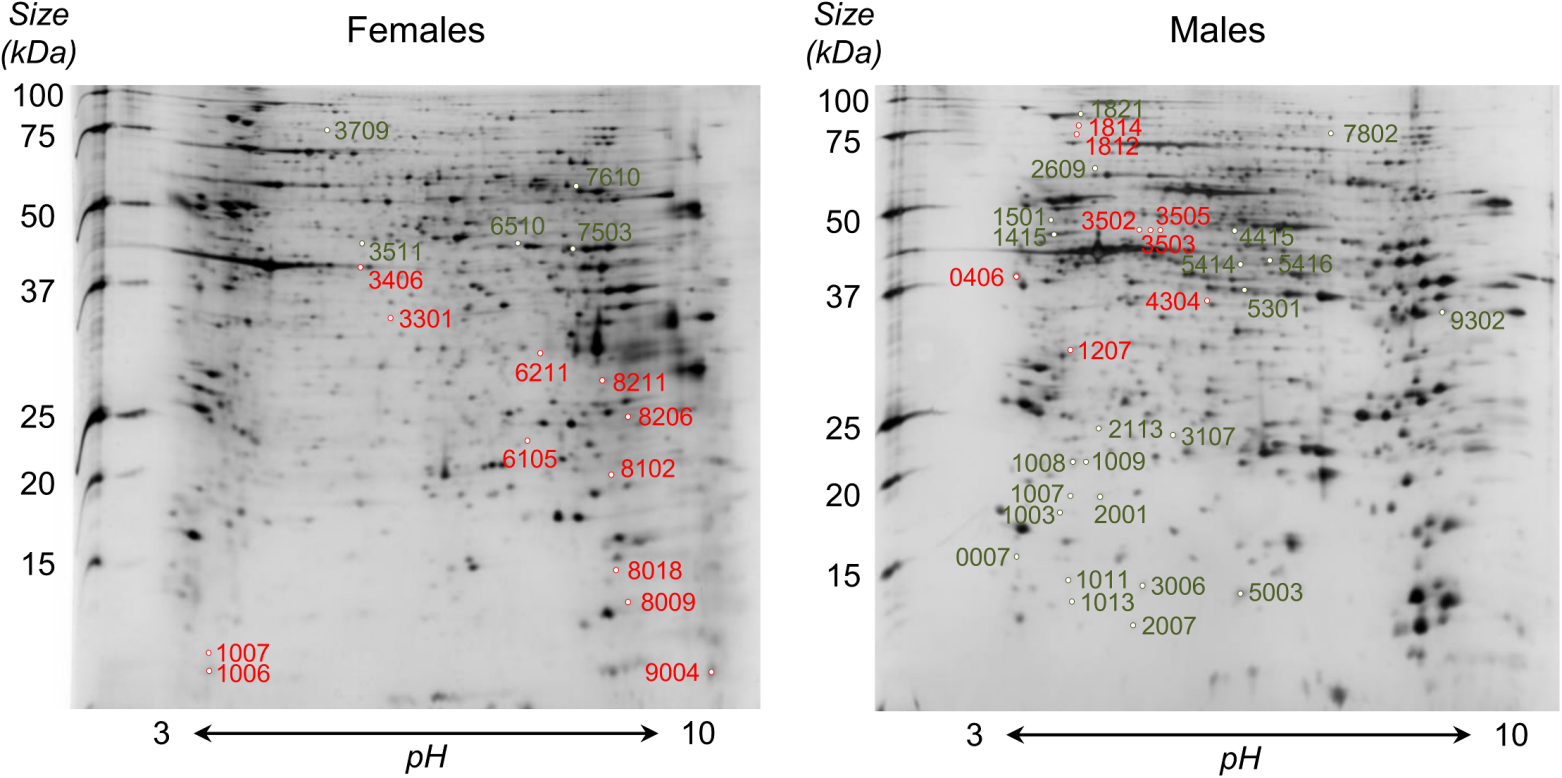
2D gels of adult females (left) and males (right) of *Schistosoma mansoni*. For each sex, spots over-expressed in latex-exposed group as compared to control group are indicated in red while under-expressed ones are represented in green. Protein identification of spots by MS/MS is available in S3 Table.

A total of 926 and 757 spots were identified in protein gels and included in the analysis from adult males and females, respectively (Fig 5). Seventeen spots were found differentially expressed (from -4.17 to 7.14 fold) between females from exposed and control groups, of which 16 provided significant matches against *S*. *mansoni* genome (S3 Table). For males, 31 spots were differentially expressed between conditions (from -12.50 to 21.98 fold) and proper protein identification was obtained for 29 of them (S3 Table).

We detected several proteins among male and female parasites associated with the schistosome muscle layer, such as the troponin T, tropomyosin, putative 22.6kDa tegument-associated antigen, antigen Sm21.7 and putative calcineurin. Additionally, our proteomic data showed three spots of putative hsp70-interacting protein (Smp_062420.1) that were detected only in male worms derived from latex exposed cercariae.

### Common functions changed in female and male worms: energy production, muscle contraction, RNA processes and amino acid metabolism

To identify Gene Ontology (GO) categories of genes and proteins differentially expressed between latex-exposed and control groups for male and female adult worms, enrichment analysis was performed. The main biological process categories specifically affected in females were “cytoskeleton/actin organization” (containing the terms “sequestration of actin monomers” and “Arp2/3 complex-mediated actin nucleation”) and “apoptotic process” (containing the term “positive regulation of apoptotic process”). Categories that were specifically affected in male worms were iron metabolism (containing the terms “intracellular sequestering of iron ion” and “iron ion transport”) and “cell division and organization” (containing the terms “mitotic spindle organization”, “phospholipid translocation” and “microtubule-based process”) (S4 Table).

Four categories were affected in both males and females which are “RNA processes”, “amino acid metabolism”, “energy process” and “muscle contraction”. Among them, the terms “regulated of muscle contraction”, “glycolytic process” and one step of biogenesis of RNA were affected in both sexes (S4 Table).

Female worms displayed less terms of “RNA processes” and “amino acid metabolism” than males (S4 Table). Male worms had 6 terms in the “amino acid metabolism” category and 3 in RNA processes while there was only two for both categories in females. Male worms showed several changes in “amino acid metabolism” and among the GO terms involved in “translational frameshifting”, “positive regulation of translational termination”, “positive regulation of translational elongation”, “protein import into mitochondrial matrix”, “protein folding” and “translational initiation” (S4 Table).

### Functional protein-association networks based on compiled available experimental evidence were conducted on both transcriptomic and proteomic data that we generated

**Fig 6:**
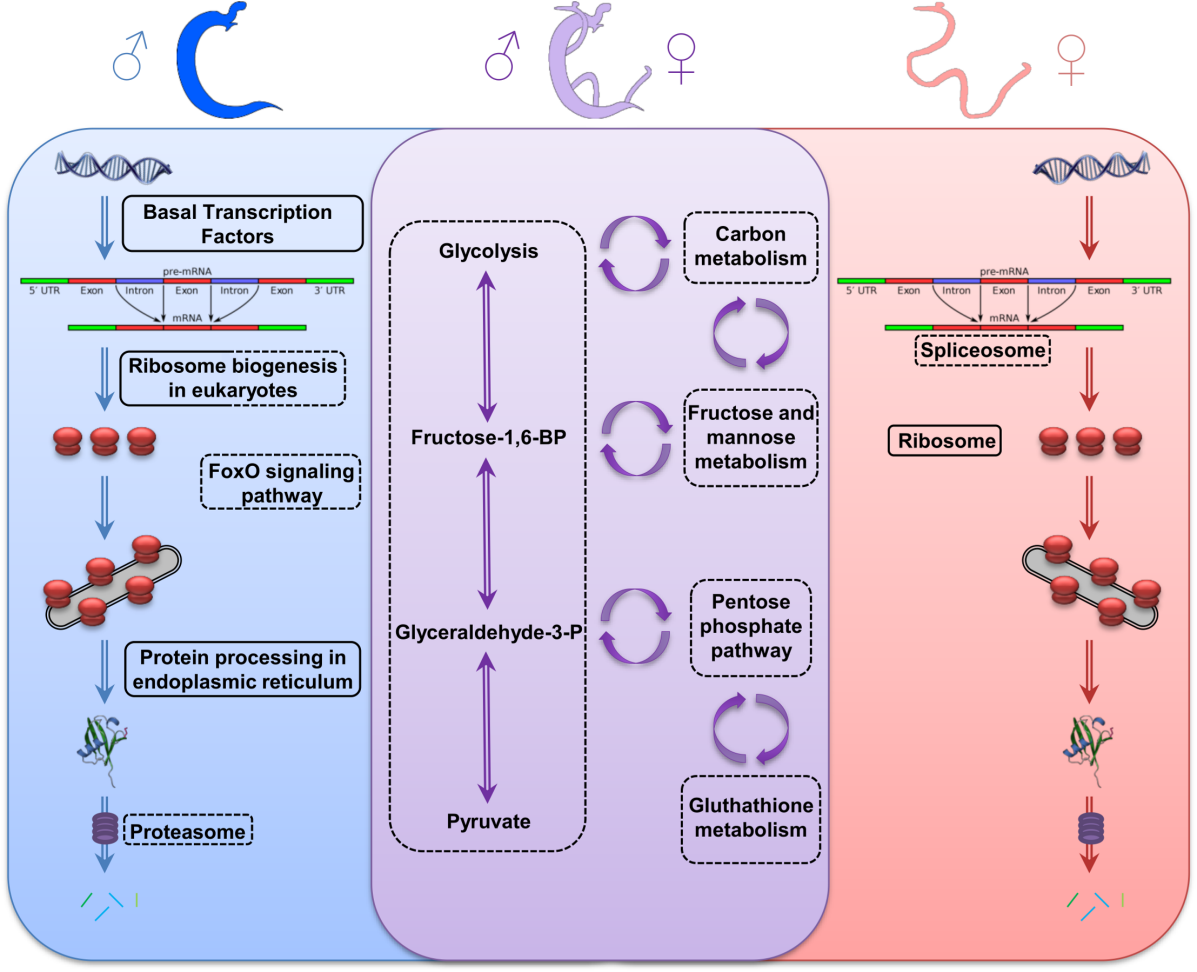
Pathways affected in *Schistosoma mansoni* adult worms after exposure to latex at cercarial stage. Anabolic and catabolic pathways affected only in male (left, blue), only in female (right, red) or in both sexes (middle, purple). Pathways down-expressed are circled by a dotted lines and pathways over-expressed are circled with full lines.

The anabolic metabolism of female adult parasites displayed reduction on “spliceosome” (observed gene count: 18) and “biosynthesis of amino acids” (observed gene count: 8) pathways. In the meantime, males displayed reduction on “ribosome biogenesis in eukaryotes” (observed gene count: 21), “biosynthesis of amino acids” (observed gene count: 5), “oxidative phosphorylation (observed gene count: 4) and “proteasome” (observed gene count: 22), but “basal transcription factors” (observed gene count: 13), “ribosome biogenesis in eukaryotes” (observed gene count: 16) and “protein processing in endoplasmic reticulum” (observed gene count: 7) pathways were increased (Fig 6; S5 Table).

The catabolic process exhibited similar patterns of reduction in both sex, especially for “glycolysis/gluconeogenesis”, “carbon metabolism”, “fructose and mannose metabolism”, “metabolic pathways” and “microbial metabolism in diverse environments” pathways. Additionally, some affected pathways were sex-specific, such as “FoxO signaling” pathway (observed gene count: 3) in male parasite (Fig 6; S5 Table).

## Discussion

We describe here the influence of a water-soluble compound used as molluscicide on the development of adult parasite worm. Molluscicides are recommended by the World Health Organization for the development of effective and practical measures for the control of schistosomiasis transmission through the elimination of snails [30]. In the snail intermediate hosts, low doses of *E*. *milii* latex affects carbohydrate metabolism and nitrogen products [11]. However, it does not impact *S*. *mansoni* cercariae survival and infection success [15]. Here we show that transient exposure of cercariae to latex decreases worm fitness and lead to severe phenotypic changes that can be observed sixty days after the treatment. The input of soluble macromolecules and/or environmental factors in the free larval stages must result in changes in gene expression which modify the phenotype and thus affect the parasite’s biology in ways that is stable over the parasite’s life span [17,19,31].

Our integrated transcriptomics, proteomics and ultrastructural analysis converge towards a scenario in which a short latex contact at larval stage has a lasting schistosomastatic action affecting the development into adult worms. Several studies reported that congruency between omics data and phenotypic features are difficult when analyzed independently [32, 33]. Transcriptome analysis gives insights into the dynamic expression of genes while proteomic takes into account all post-transcriptional and post-translational events to quantify proteins abundance, which is influenced by proteins half-life. This is why it is often difficult to predict the abundance of proteins based only on the patterns of gene expression, and *vice-versa*. Functional annotations are indicated for integrative approaches using heterogeneous datasets to improve the understanding of a determined biological event. In this sense we focused our analysis on GO annotations of transcripts/proteins differentially expressed for each sex and for each exposure group to improve the understanding of similarities in anabolic and catabolic pathways.

Energy metabolism is particularly interesting to understand this model, especially in helminths parasites that inhabit or encounter hypoxic or anoxic habitats as Schistosomes. During the aquatic environment, cercariae presents intense aerobic metabolism in the tail when it is seeking the definitive host however schistosomulum and adult worms exhibits anaerobic metabolism inside the definitive host [34]. In the present study, several steps in energy metabolism had decreased in both sexes besides modulate catabolic pathways in different metabolic chokepoints such as glyceraldehyde-3-phosphate dehydrogenase (Smp_056970.3), phosphoglycerate kinase (Smp_018890) and enolase (Smp_024110). The latex effect on metabolic chokepoints could be critical in the energy required for *Schistosoma* maturation since in these metabolic steps are expected that the inhibition of an enzyme that consumes a unique substrate or that produces a unique product can potentially toxic or cripple to essential cell functions. In addition, other important chokepoint enzyme was detected in the fatty acid biosynthetic process: 3 oxoacyl (acyl carrier protein) reductase (Smp_042680). The inability to generate long chain fatty acids or 'de novo' cholesterol by *Schistosoma* adult worms [35] requires the parasite to incorporate lipids from the host and although also involved in energy metabolism as the previous chokepoints fatty acids has an important role in female egg production. Thus, it is believed that chokepoint enzymes might be vital to the parasite and are consequently potential drug targets. It should be noted that the impairment of lipid incorporation is the basis of the single vaccine currently in clinical phase for schistosomiasis [36].

Moreover, the under-expression of other catabolic pathways (carbon metabolism, fructose and mannose metabolism, pentose phosphate pathway, glutathione metabolism) and anabolic pathways (spliceosome, cytoplasmic ribosomes, foxO signaling, oxidative phosphorylation, biosynthesis of amino acids and proteasome) forced a compensatory mechanism in an attempt to maintain the parasite homeostasis (basal transcription factors, RER associated ribosomes, protein processing in RER/Golgi). The effect in the energy availability generated strong consequences in the parasite’s development and maintenance inside the mammalian host [37].

The parasite development undergoes in functional terms related to muscle contraction, cell cycle and tegument renewed in both genres but mainly males showed morphological malformations of spines and tubercles and a complete loss of spines inside gynecophoral channel. The *S*. *mansoni* tegument is a multifunctional structure and presents vital importance for adult parasites [38]. Due to its interface with the definitive host, this structure is intimately associated to the immune system of the host. The parasite's tegument presents a constant renewal process in the outer syncytium zone [39,40] and when exposed to anthelmintic drugs (Hicantone, Oxamniquine, Praziquantel) some local deformation such as wrinkling, erosion and loss of tubers are observed [39,41]. Recent studies suggested that these parasites can use distinct populations of neoblast-like cells to in response to a variety of external stimuli [42]. Indeed, in the present work, we observed different genes/proteins and pathways related to the tegument and notably involved in tegument renewal, including bruno-like RNA binding protein [43].

Our hypothesis is that latex affects several steps of energy metabolism, including catabolic and anabolic pathways, amino acid metabolism, cell cycle and motor activities. As observed with snails, in our model the effects started at the cercariae stage and were mitotically heritable directing significant changes in phenotype and fitness over the parasite’s life span. Furthermore, the significantly reduce on reproductive activity of adult parasites, size of hepatic granuloma and the effect on sexual proportion are important factors to morbidity, transmission and maintenance of the schistosomiasis in a specific area [44, 45, 46, 47].

In summary, 60 days post exposure of cercariae to latex, it is possible to detect in the adults its impact on gene expression and protein abundance, and on the adult parasite’s phenotype and fitness. We believe that this particularly interesting illustration of the G x E concept [48] that we have recently extended towards a systems view of environment and genetic and non-genetic inheritance [49]. In our case, the effect of latex on the adult is not a direct toxicity but must trigger an early change in developmental trajectory and/or perturbations in cell memory (S1 Fig). *Bona fide* candidate for such cellular memory are circulating neoblasts. Based on the strong differences in tegumental transcripts and proteins, morphological deformations in the adult worm’s teguments, cytoskeleton/mobility differences, combined with the fact that the majority of neoblasts commit to tegument renewal [50], we believe that neoblasts are candidates for the lasting latex effect. Damage of neoblast commitment or their migration capacity would lead to inefficient tegument renewal. Since correct formation of the gynecophoral canal and muscle contractions are needed to maintain the couples, we can infer that fragility associated to the absence of spines inside of gynecophoral channel can alter the male's ability to physically maintain the female and exchange biochemical signals necessary for female maturation. This would affect the reproductive act, and cell mobility might also alter the migratory capacity necessary for oviposition, thus leading to the low number of eggs observed. Less eggs would require less energy production, which could explain the decrease in catabolic pathway associated RNAs and proteins.

The biological cycle of digenetic parasites has one of the key points of interruption the vulnerability of larval forms in limnic environments. In these environments, the larvae are affected by different soluble compounds that can significantly alter the life cycle. To date, there are few studies evaluating new molluscicides from plant extracts and its interference in the cycle of *S*. *mansoni*. The present study shows the double impact of molluscicide *E*. *milii* affecting the morphology, physiology and fitness of adult parasite worms.

In this scenario, the effects of *E*. *milii* latex differ partially on both genera in account of biological differences between them. Adult male parasite exhibits high activity in genes involved with the regulation of transmembrane transport and muscular layer. At this stage the worms' tegument is exposed to host immune cells which requires a constant renewal while it is involved in the input of molecules. On the other hand female parasite are located within the gynechephoral channel with a partial contact with host immune response. At adult stage, female worms' demand high abundance of transcripts to energy production to the production of hundreds of eggs daily. Although the same stress factor was performed on both sexes, the impact on functional annotation changed between male and female.

This study opens perspectives for a new concept in the control of schistosomiasis in endemic areas using water soluble products at lower concentrations for snails, which may also reduce parasitic load in the final host. This indirect action is inexpensive, ecological and efficient and may in the future help the antihelminthic therapy currently recommended by the WHO, increasing the use of *E*. *milii* latex from molluscicide to schistosomiastatic.

## Supporting information

S1 Fig Model for mechanism of long lasting latex effects. (TIF)

S1 Table XLOC locus and corresponding TCONS numbers for male and female transcriptomic data. (XLS)

S2 Table Genes significantly differentially expressed between control and latex-exposed *S*. *mansoni* in adult males and females. (XLS)

S3 Table Proteins significantly differentially abundant between control and latex-exposed *S*. *mansoni* in adult males and females. (XLS)

S4 Table Gene Ontology (GO) term significantly enriched between control and latex-exposed *S*. *mansoni* in adult males and females, based on transcriptomic and proteomic data. (XLS)

S5 Table Pathways significantly differentially abundant between control and latex-exposed *S*. *mansoni* in adult males and females. (XLSX)

S1 File Cuffmerge file on adult female merged transcripts. (GFF)

S2 File Cuffmerge file on adult male merged transcripts. (GFF)

S3 File Cuffmerge file on both sex merged transcripts. (GFF)
