# Supplementary figures and images for: Double impact: natural molluscicide for schistosomiasis vector control also impedes development of Schistosoma mansoni cercariae into adult parasites
Source: PLoS Negl Trop Dis. 2017 Jul 28;11(7):e0005789. doi: 10.1371/journal.pntd.0005789 (PMC5550001; doi:10.1371/journal.pntd.0005789)

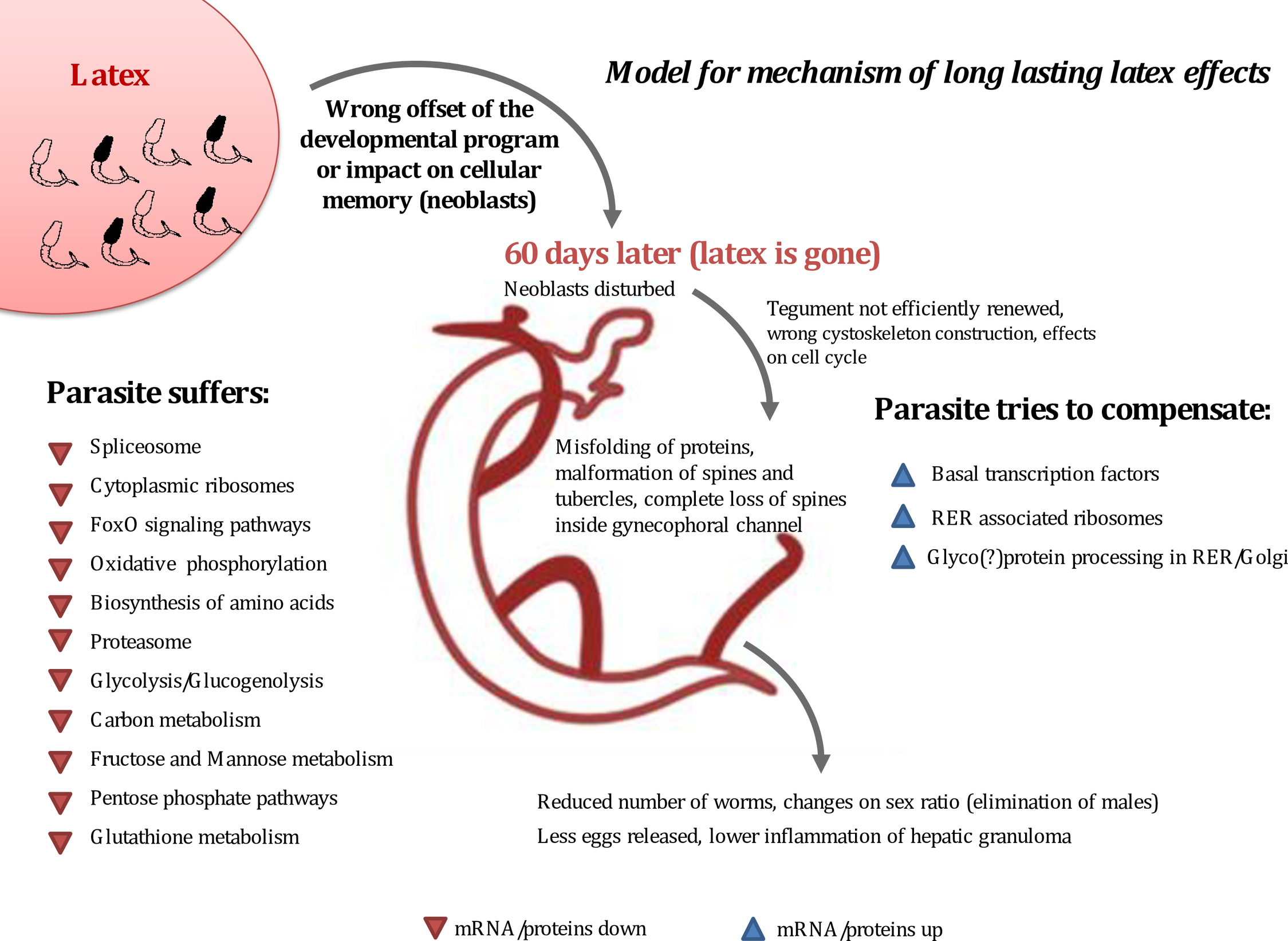

Supplement: S1 Fig — (TIF) [file pntd.0005789.s001.tif]
